# Metabolome and Microbiota Analysis Reveals the Conducive Effect of *Pediococcus acidilactici* BCC-1 and Xylan Oligosaccharides on Broiler Chickens

**DOI:** 10.3389/fmicb.2021.683905

**Published:** 2021-05-28

**Authors:** Yuqin Wu, Zhao Lei, Youli Wang, Dafei Yin, Samuel E. Aggrey, Yuming Guo, Jianmin Yuan

**Affiliations:** ^1^State Key Laboratory of Animal Nutrition, College of Animal Science and Technology, China Agricultural University, Beijing, China; ^2^NutriGenomics Laboratory, Department of Poultry Science, University of Georgia, Athens, GA, United States

**Keywords:** *Pediococcus acidilactici*, Xylan oligosaccharides, metabolome, microbiota, broiler

## Abstract

Xylan oligosaccharides (XOS) can promote proliferation of *Pediococcus acidilactic* BCC-1, which benefits gut health and growth performance of broilers. The study aimed to investigate the effect of *Pediococcus acidilactic* BCC-1 (referred to BBC) and XOS on the gut metabolome and microbiota of broilers. The feed conversion ratio of BBC group, XOS group and combined XOS and BBC groups was lower than the control group (*P* < 0.05). Combined XOS and BBC supplementation (MIX group) elevated butyrate content of the cecum (*P* < 0.05) and improved ileum morphology by enhancing the ratio of the villus to crypt depth (*P* < 0.05). The 16S rDNA results indicated that both XOS and BBC induced high abundance of butyric acid bacteria. XOS treatment elevated Clostridium XIVa and the BBC group enriched *Anaerotruncus* and *Faecalibacterium*. In contrast, MIX group induced higher relative abundance of *Clostridiaceae* XIVa, *Clostridiaceae* XIVb and *Lachnospiraceae*. Besides, MIX group showed lower abundance of pathogenic bacteria such as *Campylobacter*. Metabolome analysis showed that all the 3 treatment groups (XOS, BBC and MIX) showed lower concentrations of sorbitol and both XOS and BBC group had higher concentrations of pyridoxine levels than CT group. Besides, XOS and BBC groups enhanced the content of hydroxyphenyl derivatives 4-hydroxyphenylpyruvate 1 and 3-(3-hydroxyphenyl) propionic acid, respectively (*P* < 0.05). Notably, MIX group enhanced both 4-hydroxyphenylpyruvate 1 and 3-(3-hydroxyphenyl) propionic acid (*P* < 0.05). Thus, XOS and BBC may have a synergistic role to improve the performance of broilers by modulating gut microbiota and metabolome.

## Introduction

It is now clear that the gut microbiota and its metabolic activities have essential effects on health and performance of broiler chickens (Calik and Ergun, 2015). Probiotics are beneficial to the host by improving the balance of the gut microbiota (Pender et al., 2017). Dietary supplementation with probiotic *Pediococcus* (*P*.) *acidilactici* was shown to enhance gut health and growth performance of broilers (Taheri et al., 2010; Jazi et al., 2018). Besides, *P. acidilactici* can improve gut morphology by increasing the villus height in duodenum and ileum as well (Taheri et al., 2010). The metabolism of *P. acidilactici* bacteria produced lactic acid and secreted bacteriocins, which inhibited the growth of pathogenic bacteria such as coliforms and Salmonella (Taheri et al., 2010; Jazi et al., 2018). Broiler chickens fed a diet supplemented with *P. acidilactici* had lower coliforms numbers in the ileum than that of their control counterparts (Taheri et al., 2010). Therefore, dietary supplementation with *P. acidilactici* effectively protected birds against the adverse growth effects associated with pathogens (Lee et al., 2007).

Probiotics activity have been shown to be augmented by consuming prebiotics (Gibson et al., 2004). Although xylan oligosaccharides (XOS) cannot be degraded by gut digestive enzymes, it can produce short-chain fatty acids (SCFA) by gut microbial fermentation (Kabel et al., 2002). XOS can be utilized by *Bifidobacterium* spp. and *Lactobacillus* spp. (Moura et al., 2007) resulting in greater production of lactate, butyrate and increased *Bifidobacterium* and *Lactobacillus* populations (De Maesschalck et al., 2015; Pourabedin et al., 2015; Moniz et al., 2016; Yuan et al., 2018). In addition, XOS can improve feed conversion ratio (De Maesschalck et al., 2015; Ribeiro et al., 2018) and increase ileum villus length (De Maesschalck et al., 2015). Though XOS can be used by probiotic strains such as *Lactobacillus* spp. and *Bifidobacterium* spp., it is not degraded by enteric pathogens such as *Staphylococcus aureus*, *Clostridium difficile*, *Salmonella enterica*, and *Campylobacter jejuni* (Moura et al., 2007; Kondepudi et al., 2012). Besides, dietary XOS also stimulated immune response against infection and reduced *Salmonella* colonization (Pourabedin et al., 2016). Therefore, XOS modulates animal performance enhancement by improving feed digestion, feed intake and triggering the shift of the microbiome to a more favorable composition (Ribeiro et al., 2018). Our previous study also found supplemental combination of xylanase and wheat arabinoxylan-specific debranching enzymes enhanced XOS level in the intestine, which consequently benefited growth performance and gut health in broiler chickens (Lei et al., 2016).

It has been demonstrated through *in vitro* studies that XOS could promote the proliferation of *P. acidilactici* BCC-1, which harbors genes with carbohydrate transport and metabolism functions (Lei et al., 2018). However, the mechanism through which *P. acidilactici* BCC-1 improves growth performance and gut health is yet to be elucidated. Developments in omic science have led to significant improvements in the understanding of the biological mechanisms that underlie phenotypes (Aggrey et al., 2019). The metabolome can identify several host tissue and microbiota molecules alluding to plausible interaction between the host and its microbiome (Marcobal et al., 2013; Aggrey et al., 2019). Thus, our study aimed to investigate whether dietary supplementation of *P. acidilactic* BCC-1 and XOS has a synergistic role in broilers’ gut health and growth performance, and it if associated with the alternations of cecal microbiota and microbial metabolism.

## Materials and Methods

### Materials

XOS (extracted from corncob, 95% purity, DP of 2-7 and containing 95.6% XOS, 2.4% xylose, 1.4% glucose, 0.5% arabinose, and 0.1% raffinose) was obtained from Shandong LongLive Biotechnology (DeZhou, Shandong, China). *P. acidilactici* BCC-1 was isolated from the cecum of 36-day-old broiler without feeding any antibiotics (Lei et al., 2018).

### Experiment Design and Bird Management

This study was conducted in an experimental chicken farm of the College of Animal Science and Technology, China Agricultural University. A total of 480 one-day-old male Arbor Acres plus chicks were randomly divided into 4 treatment groups. There were 8 replicates per treatment and 15 chicks per replicate. The treatments include 1) control group (CT): basal diet without XOS or *P. acidilactici* BCC-1; 2) XOS: basal diet plus 0.15% XOS; 3) BBC: basal diet plus 10^9^ cfu/kg *P. acidilactici* BCC-1; 4) MIX: basal diet plus 0.15% XOS and 10^9^ cfu/kg *P. acidilactici* BCC-1. The feed was formulated to meet the standards of nutrient requirements (National Research Council [NRC], 1994) without antibiotics. The composition of the basal diet and nutritional levels are shown in Table 1. Chickens were raised from 1 to 21 days of age, and feed and water were provided *ad libitum*. The light regimen was 23L:1D. The birds were raised at 32°C for the first week and then decreased by 3°C per week until 26°C.

**TABLE 1:** Composition and nutrient levels of diets (%).

**Item**	**Content**
**Ingredients**	
Wheat	64.53
Soybean meal	28.52
Soybean oil	2.89
Dicalcium phosphate	1.97
Limestone	0.93
Sodium chloride	0.35
Choline chloride (50%)	0.25
L-Lys HCl	0.17
Trace mineral^*a*^	0.20
DL-methionine (Met)	0.14
Ethoxyquin	0.03
Vitamin premix^*b*^	0.03
Total	100.00
**Calculated composition**	
ME, Mcal/kg	2.90
CP	21.50
Available Phosphorus (*P*)	0.45
Calcium (Ca)	1.00
Lysine	1.10
Met + Cysteine (Cys)	0.93
Threonine	0.82

*^*a*^The trace minerals supplied the following per kilogram of feed: copper, 8 mg; zinc, 75 mg; iron, 80 mg; manganese, 100 mg; selenium, 0.15 mg; and iodine, 0.35 mg. ^*b*^The vitamin premix supplied the following per kilogram of complete feed: vitamin A, 12 500 IU; vitamin D_3_, 2500 IU; vitamin K_3_, 2.65 mg; vitamin B_1_, 2 mg; vitaminB_2_, 6 mg; vitamin B_12_, 0.025 mg; vitamin E, 30 IU; biotin, 0.0325 mg; folic acid, 1.25 mg; pantothenic acid, 12 mg; and niacin, 50 mg.*

### Sample Collection

On 21-day old, after 5 h of starvation, 1 bird per replicate was randomly selected for sampling. The birds were anesthetized by an injection of sodium pentobarbital (30 mg/kg body weight) in the wing vein followed by jugular exsanguination. Two more birds are randomly selected in each group for metabolome analysis (*n* = 10). The cecal content of each broiler was collected and put into liquid nitrogen immediately, then stored at −80°C until further analysis. Samples of each intestinal tract including duodenum, jejunum and ileum were taken for the determination of gut morphology. Digesta samples for viscosity analysis were collected from the jejunum. Chyme samples for pH determination were collected from the distal part of the ileum and cecum. The digesta viscosity was determined by using an Obarma’s viscosimeter as described by previous study (Lei et al., 2016).

### Intestinal Morphology of Broilers

Intestinal samples including duodenum, jejunum and ileum were fixed with paraformaldehyde. After staining, various intestinal morphological indexes including muscular layer thickness, villus height, crypt depth and the ratio of villus height to crypt depth (V/C) were determined. For the measurement of each intestinal segment, cross-sections of 10 villi and 10 crypts were measured per tissue sample.

### Chemical Analyses of SCFA

Procedures for SCFA analysis were described by a previous study (Williams et al., 2011). The gas chromatography GC-17A (Shimadzu, Kyoto, Japan) with a flame ionization detector (FID) equipped with a DB-FFAP column (30 m × 0.53 mm) (J&W Scientific) was applied to detect SCFA.

### Statistical Analysis

One-way ANOVA and Duncan’s multiple comparisons were applied when a significant difference was observed among groups by using SPSS Version 18.0. Statistical significance was considered when *P* < 0.05. Results were given as the mean ± standard error of the mean.

### Metabolome Determination of Cecal Chyme

The replicates of metabolome are *n* = 10 for all groups. The cecal chyme metabolomic content was determined by Shanghai Biotree Biotech Co., Ltd. (Shanghai, China). The internal standard L-2-chlorophenylalanine (CAS#: 103616-89-3, ≥ 98%) was bought from Hengbai Biotechnology Co., Ltd. (Shanghai, China) and derivatization reagent BSTFA (including 1% TMCS, v/v) was purchased from REGIS Technologies Inc. (Morton Grove, IL, United States). An Agilent 7890 gas chromatograph system combined with a Pegasus 4D time-of-flight mass spectrometer (LECO Corp, St. Joseph, MI, United States) was used for GC/TOFMS analysis. The DB-5MS capillary column was applied in this system (30 m × 250 μm inner diameter, 0.25 μm film thickness (J&W Scientific, Folsom, CA, United States). Analyte injection volume was one μL with splitless mode. The carrier gas was helium, the purge flow at the front inlet was 3 mL/min, and the gas flow rate through the column was 1 mL/min. The initial temperature was kept at 80°C for 1 min, then raised to 290°C at a rate of 10°C/min, then held at 290°C for 12 min. The temperatures in injection, transfer line, and ion source were 280, 295, and 220°C, respectively. In the electron impact mode, the energy was −70 eV. After a 7 min delay in the solvent, mass spectrometry data were acquired at a rate of 12 spectra per second in full-scan mode with the m/z range of 50–600.

### Metabolome Data Analysis

Chroma TOF 4.3X software and LECO-Fiehn Rtx5 database (LECO Corp, St. Joseph, MI, United States) were used to extract raw peaks, filter and calibrate data baselines, align peak, analyze deconvolution, identify peak and integrate the peak area (Kind et al., 2009). Peaks were identified by retention time index (RI), with a RI tolerance of 5000. The obtained three-dimensional data including the peak number, sample name, and normalized peak area were processed by SIMCA14.1 software package (V14.1, MKS Data Analytics Solutions, Umea, Sweden) for principal component analysis (PCA) and orthogonal projections to latent structures-discriminate analysis (OPLS-DA). In OPLS-DA model, the first principal component of variable importance in the projection (VIP) values exceeding 1.0 are most relevant and were first selected as changed metabolites. The remaining variables were then assessed by Student’s t-test (*P*-value <0.05). In addition, databases including KEGG^1^ and NIST^2^ were applied to identify the metabolites involved pathways. MetaboAnalyst^3^ also used for pathway analysis.

### 16S rDNA Sequencing

The replicates of 16S sequencing are *n* = 6 for CT and XOS group and *n* = 7 for BBC and MIX group. The extraction of bacterial DNA was conducted as described by our previous study (Wu et al., 2018). Briefly, DNA was extracted from 180–220 mg of the cecal samples using a QIAampTM Fast DNA Stool Mini Kit (Qiagen, cat# 51604) according to the manufacturer’s instructions. Total DNA was quantified using a Thermo NanoDrop 2000 UV microscope spectrophotometer and 1% agarose gel electrophoresis. The 16S rDNA high-throughput sequencing was conducted by Realbio Genomics Institute (Shanghai, China) through an Illumina Hiseq PE250 platform. The universal primers 341F (CCTACGG GRSGCAGCAG) and 806R (GGACTACVVGGGTATCTAATC) were used to amplify the V3-V4 region of the 16S rDNA gene. The raw pair-end reads were merged and quality-filtered by using PANDAseq (v2.9) to remove tags with lengths <220 nt, an average quality score of <20, and tags containing >3 ambiguous bases (Masella et al., 2012). The USEARCH (v7.0.1090) in QIIME software was applied to cluster the quality-filtered sequences into 97% operational taxonomic units (OTUs). Each OTU was classified by the Ribosomal Database Project (RDP) algorithm trained on the Greengenes database.^4^ The alpha diversity was determined by the software package QIIME.^5^

### 16S rDNA Analysis

The Wilcoxon rank sum test was used to assess alpha diversity among the four treatment groups. Venn diagrams were built by Venn Diagram package in R v3.1.0. Principal component analysis (PCA) was applied to evaluate the relationships between samples based on the composition of the microbiota (Sanders et al., 2015). A linear discriminant analysis (LDA) effective size (LEfSe) was used to further compare the relative abundance profiles of bacteria between two groups (Segata et al., 2011). Taxonomic units with a log LDA score >2 were determined to be significant differences in abundance. Phylogenetic identification of communities by reconstruction of unobserved states (PICRUSt) was applied to predict function composing of metagenomic communities based on 16S rDNA (Langille et al., 2013). The metagenomic reads were submitted to the NCBI-SRA database under accession number PRJNA592830.

## Results

### Growth Performance

The effect of XOS and *P. acidilactici* BCC-1 supplementation on the growth performance of broilers is shown in Table 2. The feed conversion ratio of BBC group, XOS group and MIX group was lower than that of the control group (*P* < 0.05). However, there was no significant difference among groups in body weight gain and feed intake.

**TABLE 2:** The effect of XOS and *Pediococcus acidilactici* BCC-1 supplementation on growth performance of broilers from 1–21 d.

**Treatment^1^**	**BWG (g)**	**FI (g)**	**FCR**
CT	621.03 ± 13.68	975.73 ± 23.94	1.57 ± 0.02^b^
XOS	621.63 ± 13.68	946.57 ± 12.04	1.53 ± 0.01^a^
BBC	628.56 ± 28.92	959.18 ± 14.33	1.53 ± 0.01^a^
MIX	649.18 ± 14.55	982.60 ± 20.52	1.51 ± 0.02^a^
*P-*value	0.151	0.166	0.028

*^a,b^Means in the same row without common superscripts are significantly different (*P* < 0.05).*

*^1^CT: Basal diet (0% XOS and 0 cfu/kg *P. acidilactici* BCC-1); XOS: Basal diet plus 0.15% XOS; BBC: Basal diet plus 10^9^cfu/kg *P. acidilactici* BCC-1; MIX: Basal diet plus 0.15% XOS and 10^9^cfu/kg *P. acidilactici* BCC-1.*

### Chyme Viscosity of Jejunum and pH Value of Ileum and Cecum

The cecum pH in XOS, BBC and MIX group were lower than CT group. Also, BBC and MIX had a lower value of chyme viscosity of jejunum than the CT group (*P* < 0.05). However, there was no statistical difference in ileum pH (Table 3). XOS supplementation had no effect on jejunum chyme viscosity, but significantly decreased the pH value of cecum (*P* < 0.05).

**TABLE 3:** The effect of XOS and *Pediococcus acidilactici* BCC-1 supplementation on pH value and chyme viscosity of broilers on d 21.

**Treatment^1^**	**pH of ileum**	**pH of cecum**	**Chyme viscosity of jejunum**
CT	5.87 ± 0.07	5.83 ± 0.18^b^	1.51 ± 0.02^b^
XOS	6.27 ± 0.22	5.18 ± 0.12^a^	1.52 ± 0.01^b^
BBC	6.11 ± 0.1	5.12 ± 0.1^a^	1.43 ± 0.02^a^
MIX	6.11 ± 0.16	5.22 ± 0.12^a^	1.38 ± 0.03^a^
*P* value	0.284	0.004	0.002

*^a,b^Means in the same row without common superscripts are significantly different (*P* < 0.05).*

*^1^CT: Basal diet (0% XOS and 0 cfu/kg *P. acidilactici* BCC-1); XOS: Basal diet plus 0.15% XOS; BBC: Basal diet plus 10^9^cfu/kg *P. acidilactici* BCC-1; MIX: Basal diet plus 0.15% XOS and 10^9^cfu/kg *P. acidilactici* BCC-1.*

### Contents of Lactic Acid and SCFA in Cecum Chyme

The MIX treatment significantly increased the content of butyric acid in cecal chyme (*P* < 0.05). However, the addition of XOS and BBC had no effect on the content of each kind of SCFA (except butyric acid) and total acid production of the cecum (Table 4).

**TABLE 4:** The effect of XOS and *Pediococcus acidilactici* BCC-1 supplementation on the production of lactic acid and SCFA in cecum chyme of broilers on d 21 (mmol/g).

**Treatment^1^**	**Lactic acid**	**Methanoic acid**	**Acetic acid**	**Propionic acid**	**Butyric acid**	**Pentanoic acid**	**Total content**
CT	468.92 ± 118.8	72.19 ± 13.22	2555.67 ± 296.45	1850.01 ± 275.10	783.10 ± 123.08^a^	118.45 ± 19.28	5905.03 ± 477.62
XOS	887.99 ± 425.24	63.37 ± 9.52	2538.54 ± 178.08	2000.15 ± 210.76	714.71 ± 62.56^a^	111.42 ± 18.08	6390.53 ± 412.11
BBC	947.19 ± 292.64	37.69 ± 13.76	2712.74 ± 131.63	2341.76 ± 161.38	946.21 ± 75.78^ab^	101.17 ± 8.42	7145.26 ± 339.36
MIX	138.63 ± 64.75	55.46 ± 10.26	3217.53 ± 175.41	1926.60 ± 222.96	1103.85 ± 111.87^b^	132.67 ± 19.40	6669.99 ± 184.04
*P* value	0.176	0.231	0.091	0.440	0.033	0.631	0.161

*^a,b^Means in the same row without common superscripts are significantly different (*P* < 0.05).*

*^1^CT: Basal diet (0% XOS and 0 cfu/kg *P. acidilactici* BCC-1); XOS: Basal diet plus 0.15% XOS; BBC: Basal diet plus 10^9^cfu/kg *P. acidilactici* BCC-1; MIX: Basal diet plus 0.15% XOS and 10^9^cfu/kg *P. acidilactici* BCC-1.*

### Intestinal Morphology

In the duodenum, the XOS group had a higher villus height than BBC group (*P* < 0.05). In the ileum, MIX group showed a significant higher V/C ratio than CT, XOS and BBC group. Also, the MIX group had a higher ileum villus height than the BBC group. Besides, the muscular layer thickness in the MIX group was lower than CT and BBC group (Table 5).

**TABLE 5:** The effect of XOS and *Pediococcus acidilactici* BCC-1 supplementation on intestinal morphology of broilers on d 21.

	**duodenum**				**Jejunum**				**ileum**			
**Treatment**	**Villus height (μm)**	**Crypt depth (μm)**	**V/C ratio**	**Muscular layer thickness (μm)**	**Villus height (μm)**	**Crypt depth (μm)**	**V/C ratio**	**Muscular layer thickness (μm)**	**Villus height (μm)**	**Crypt depth (μm)**	**V/C ratio**	**Muscular layer thickness (μm)**
CT	975.21 ± 10.75^ab^	209.50 ± 3.67	4.68 ± 0.11	705.92 ± 14.17	1048.22 ± 24.94	218.56 ± 4.46	4.81 ± 0.11	684.91 ± 15.81	1013.26 ± 17.92^ab^	235.19 ± 7.71	4.38 ± 0.16^a^	729.21 ± 14.34^b^
XOS	1053.00 ± 28.80^b^	226.93 ± 6.21	4.66 ± 0.14	686.45 ± 11.71	1035.35 ± 34.59	217.98 ± 8.37	4.78 ± 0.17	708.35 ± 16.40	1011.92 ± 20.22^ab^	231.87 ± 6.94	4.39 ± 0.12^a^	697.63 ± 13.96^ab^
BBC	928.03 ± 24.61^a^	204.84 ± 6.35	4.57 ± 0.18	713.42 ± 39.51	977.13 ± 22.20	207.75 ± 5.62	4.73 ± 0.15	732.70 ± 22.22	970.59 ± 17.85^a^	216.73 ± 4.18	4.50 ± 0.11^a^	713.62 ± 6.97^b^
MIX	1023.27 ± 33.99^ab^	216.99 ± 7.03	4.74 ± 0.16	751.72 ± 27.53	1023.25 ± 31.10	216.50 ± 5.79	4.76 ± 0.19	682.81 ± 12.96	1056.50 ± 24.02^b^	214.25 ± 4.84	4.96 ± 0.16^b^	670.66 ± 13.16^a^
*P* value	0.006	0.056	0.888	0.334	0.323	0.570	0.986	0.156	0.047	0.055	0.028	0.015

*^a,b^Means in the same row without common superscripts are significantly different (*P* < 0.05).*

*^1^CT: Basal diet (0% XOS and 0 cfu/kg *P. acidilactici* BCC-1); XOS: Basal diet plus 0.15% XOS; BBC: Basal diet plus 10^9^cfu/kg *P. acidilactici* BCC-1; MIX: Basal diet plus 0.15% XOS and 10^9^cfu/kg *P. acidilactici* BCC-1.*

### Microbiota of Cecum Chyme

**FIGURE 1:**
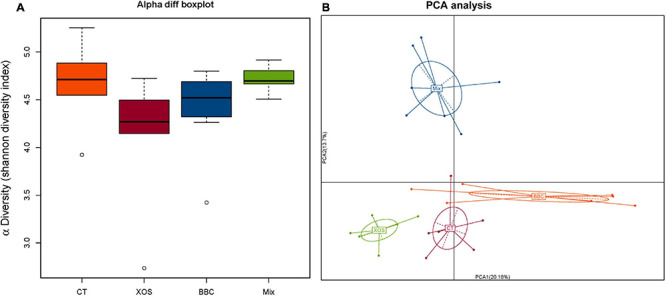
The α diversity **(A)** and Principal Component Analysis (PCA) **(B)** of cecal microbiota among four groups. CT group, control group with basal diet; XOS group, basal diet plus 0.15% XOS; BBC group, basal diet plus 10^9^ cfu/kg *P. acidilactici* BCC-1; MIX group, basal diet plus 0.15% XOS and 10^9^ cfu/kg *P. acidilactici* BCC-1.

**FIGURE 2:**
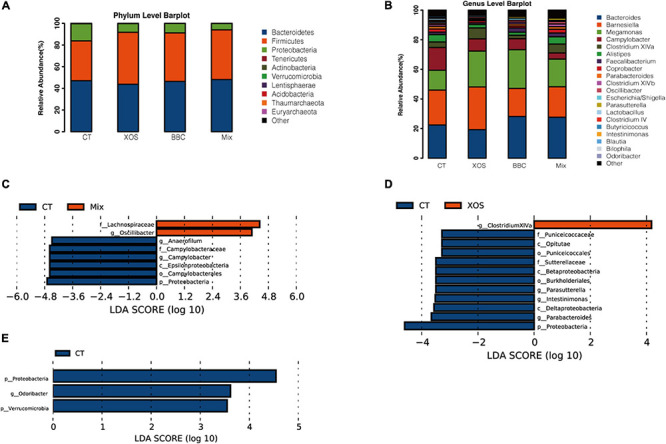
Relative abundance of the dominant bacterial communities at phylum **(A)** and genus **(B)** level and log-transformed linear discriminant analysis (LDA) scores of the significant biomarkers of MIX vs CT group **(C)**, XOS vs CT group **(D)** and BBC vs CT group **(E)**.

In PCA analysis, there is a clear separation among the four groups. Broilers fed a diet with both XOS and *P. acidilactici* BCC-1 (MIX) had lower alpha diversity than the control group. Furthermore, the MIX group showed a higher alpha diversity than either XOS or BBC groups (Figure 1). Compared with the CT group, all 3 treatment groups (XOS, BBC and MIX) showed a higher abundance of *Firmicutes* and lower *Proteobacteria* at phylum level (Figure 2A). At genus level, all 3 treatment groups showed higher abundance of *Megamonas* and lower abundance of *Campylobacter* (Figure 2B).

The LEfSe analysis showed MIX group enriched *Lachnospiraceae* and *Oscillibacter* while CT group enriched multiple pathogenic bacteria including *Campylobacter* and *Proteobacteria* (Figure 2C). The XOS group enriched butyric bacteria *Clostridiaceae* XIVa whereas pathogenic bacteria *Proteobacteria* enriched in CT group (Figure 2D). Compared with BBC group, the CT group enriched *Proteobacteria* as well (Figure 2E).

**FIGURE 3:**
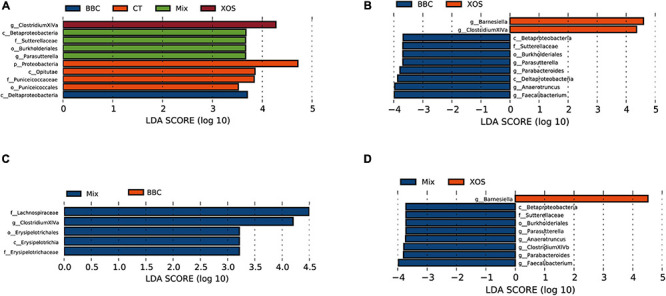
LDA scores of the significant biomarkers among four groups **(A)**, BBC vs XOS group **(B)**, MIX vs BBC group **(C)** and MIX vs XOS group **(D)**.

When compared among all groups, enrichment species in XOS group and BBC treatment groups were *Clostridium* XIVa and *Deltaproteobacteria*, respectively. The enrichment bacteria in the MIX group were β-*Proteobacteria*, *Sutterellaceae*, *Burkholderia* and *Parasutterella*. In contrast, the *Proteobacteria*, *Opitutae*, *Puniceicoccaceae* and *Puniceicoccales* were enriched in the control group (Figure 3A).

Compared with the BBC group, the XOS treatment enriched *Barnesiella* and *Clostridium* XIVa. In contrast, β-*Proteobacteria*, *Sutterellaceae*, *Burkholderiales*, *Parasutterella*, *Parabacteroides*, *Deltaproteobacteria*, *Anaerotruncus* and *Faecalibacterium* were enriched in the BBC group (Figure 3B). The MIX group enriched *Lachnospiraceae*, *Clostridiaceae* XIVa, *Erysipelotrichales*, *Erysipelotrichia* and *Erysipelotrichaceae* when compared with BBC group (Figure 3C). Compared with the XOS treated group, the MIX group enriched *Anaerotruncus*, *Parabacteroides*, *Clostridium* XIVb, *Sutterellaceae*, *Burkholderiales*, *Parasutterella*, β-*Proteobacteria* and *Fusobacterium*. In contrast, *Barnesiella*, *Streptococcus* and *Streptococcaceae* were enriched in the XOS group (Figure 3D).

Compared with the control group, microbial functional genes in XOS group were enriched in the pathways including transporters, ABC transporters, amino sugar and nucleotide sugar metabolism, galactose metabolism, pentose phosphate pathway and pentose and glucuronate interconversions (Supplementary Figure 1A). Genes related to galactose metabolism and amino sugar and nucleotide sugar metabolism were enriched in BBC group as well. BBC group enriched genes involved in glycine, serine and threonine metabolism, vitamin B_6_ metabolism, cysteine and methionine metabolism, arginine and proline metabolism and fructose and mannose metabolism pathways (Supplementary Figure 1B). For MIX group, genes were mapped on carbohydrate metabolism, transcription and xenobiotics biodegradation and metabolism pathways (Supplementary Figure 1C).

### Metabolites Composition of Cecum Chyme

**FIGURE 4:**
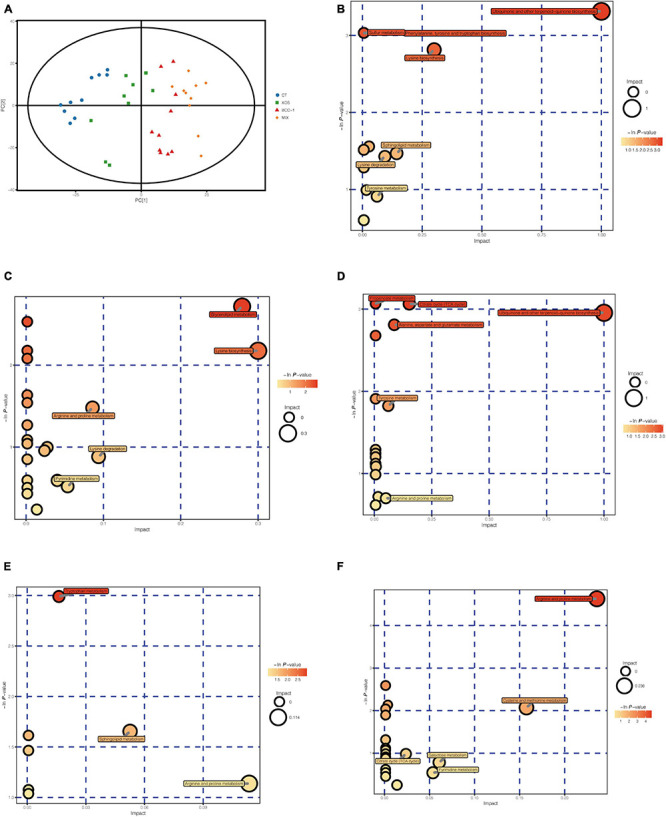
Score scatter plot for PCA model of four groups **(A)** and differential metabolites enriched metabolism pathways of XOS vs CT **(B)**, BBC vs CT **(C)**, MIX vs CT **(D)**, MIX vs XOS **(E)** and MIX vs BBC **(F)**.

The samples from each treatment group are well-differentiated and all samples are within the 95% confidence interval (Hotelling’s T-squared ellipse), indicating a clear difference in cecal chyme among the 4 treatment groups (Figure 4A). We have detected a total of 550 effective peaks and flited by quality control to retain 498 metabolites.

There were 12 metabolites that were significantly different between XOS and CT treatment groups (Supplementary Appendix Table 1). Compared with the CT group, XOS groups showed higher concentrations of 4-hydroxyphenylpyruvate 1, *O*-acetylserine 1, pyridoxine and glycine-proline while has lower contents of sorbitol, gluconic acid and salicylaldehyde. In contrast, BBC group has higher concentrations of myo-inositol, purine riboside, 2-hydroxy-3-isopropylbutanedioic acid, 3-(3-hydroxyphenyl) propionic acid, *O*-acetylserine 1 and pyridoxine whereas has lower levels of succinic acid, citramalic acid, 3,4-dihydroxybenzoic acid, xanthine and various carbohydrates such as sorbitol, xylose 1, D-arabitol and lyxose 1when compared to the CT group (Supplementary Appendix Table 2). Interestingly, both the XOS and BBC groups showed higher concentrations of O-acetylserine 1 and pyridoxine while had lower concentrations of sorbitol, salicylaldehyde, palmitoleic acid and gluconic acid 1 when compared with the CT group.

There were 32 metabolites there were significantly different between the MIX and CT treatment groups (Table 6). The concentrations of allo-insoitol and 4-hydroxyphenylpyruvate 1 in the MIX group was massively higher than in the CT group. Some other higher metabolites include elaidic acid, 3-(4-hydroxyphenyl) propionic acid, 3-(3-hydroxyphenyl) propionic acid and inosine. MIX group has lower concentrations of pentadeconic acid, succinic acid, citramalic acid, salicylaldehyde and multiple carbohydrates including sorbitol, xylose 1, cellobiose 1, fucose 1, D-arabitol and lyxose 1 than the CT group. It should be pointed out that all the treatment groups (XOS, BBC and MIX) have lower concentrations of sorbitol, palmitoleic acid and salicylaldhyde than control group.

**TABLE 6:** Differential metabolites in MIX and CT group.

**Metabolites**	**VIP**	***P* value**	**Fold change**
Allo-inositol	1.71	0.044	25.03
4-Hydroxyphenylpyruvate 1	1.61	0.016	15.31
Elaidic acid	1.29	0.001	4.80
3-(4-hydroxyphenyl) propionic acid	2.09	0.0005	3.94
3-(3-hydroxyphenyl) propionic acid	2.01	0.0009	3.25
2-Hydroxyvaleric acid	1.91	0.015	2.55
N-Carbamylglutamate 4	1.17	0.025	2.34
Alpha-Aminoadipic acid	2.03	0.0001	2.30
Indole-3-acetamide 2	1.92	0.0004	2.28
Inosine	1.30	0.031	2.18
Levoglucosan	1.75	0.021	0.29
Cellobiose 1	1.12	0.048	0.27
Fucose 1	1.67	0.007	0.24
Trans-4-hydroxy-L-proline 2	1.25	0.002	0.22
3-Methylglutaric Acid	1.80	0.002	0.17
N-Acetyl-beta-D-mannosamine 3	1.25	0.010	0.09
Stearic acid	2.47	0.0005	0.039
4-Hydroxyphenylacetic acid	2.26	0.0002	0.036
Xylose 1	2.17	0.029	3.09E-07
Oleic acid	2.27	0.023	2.24E-07
Sorbitol	1.72	0.017	2.10E-07
Salicylaldehyde	1.72	0.030	1.88E-07
Palmitoleic acid	2.42	0.005	1.67E-07
2-Deoxyerythritol	2.04	0.003	1.40E-07
Cis-Phytol	2.73	0.0002	1.13E-07
D-Arabitol	2.50	0.012	8.23E-08
Lyxose 1	1.86	0.028	6.96E-08
3-Hydroxy-3-methylglutaric acid	2.24	0.035	4.77E-08
3,4-Dihydroxybenzoic acid	2.49	5.32E-05	4.69E-08
Citramalic acid	1.88	0.009	3.23E-08
Succinic acid	1.81	0.030	1.48E-08
Pentadecanoic acid	2.54	0.0006	8.68E-09

*VIP, Variable Importance in Projection; *P* value calculated by Student’s t test; Fold Change >2 or ≤0.3. If Fold change >1, it means that this metabolite is higher in the MIX group than that in the CT group; Vice versa.*

Compared to the XOS group, the MIX group showed higher concentrations of 5-hydroxyindole-3-acetic acid (over 3 million-fold higher), 2-butyne-1,4 diol, 2-butyne-1,4-diol and DL-p-hydroxyphenyllactic acid while has lower level of 3,4-dihydroxybenzoic acid and pentadecanoic acid (Supplementary Appendix Table 3). Supplementary Appendix Table 4 shows the comparison between the BBC and MIX treatment groups. MIX group has almost 3 million folds higher level of gluconic acid was than BBC group. Other notable metabolites that were significantly higher in the MIX group were xanthine, pantothenic acid and urea. Compared with BBC group, MIX group has lower concentrations of pentadecanoic acid and multiple carbohydrates such as fucose 1, sophorose 2 and isomaltose 2.

From the pathway enrichment analysis, ubiquinone and other terpenoid-quinone biosynthesis, phenylalanine, tyrosine and tryptophan biosynthesis, sulfur metabolism, lysine biosynthesis, sphingolipid metabolism, lysine degradation and tyrosine metabolism were enriched in the XOS treatment group compared with the CT group (Figure 4B). The pathways enriched in the BBC group compared with CT group include lysine biosynthesis, arginine and proline metabolism, lysine degradation and pyrimidine metabolism (Figure 4C). The major pathways enriched in the MIX group compared with the CT group are ubiquinone and other terpenoid-quinone biosynthesis, propanoate metabolism, citrate cycle, alanine, aspartate and glutamate metabolism, tyrosine metabolism and arginine and proline metabolism (Figure 4D). The major enriched pathways between the XOS and MIX group were tryptophan metabolism, sphingolipid metabolism and arginine and proline metabolism (Figure 4E) and that between MIX and BBC groups were arginine and proline metabolism, cysteine and methionine metabolism, galactose metabolism and pyrimidine metabolism (Figure 4F).

## Discussion

In the current study, the FCR in the XOS, BBC and MIX groups were lower than the control group, indicating that the dietary supplementations improved feed efficiency. The concomitant reduction in cecal pH and decreased jejunal chyme viscosity after XOS and BBC supplementation points to improved gut fermentation. Our previous study has shown that *P. acidilactici* BCC-1 proliferated when given with XOS (Lei et al., 2016). Therefore, BBC and XOS may play a synergistic role. In the current study, the MIX treatment increased the content of butyric acid and improved gut morphology by increasing the V/C ratio of ileum. The muscular layer thickness of ileum was significantly lower than CT as well. A previous study found wetting diets based on cereal grains caused decreased viscosity of gut contents, which accompanied by the reduced thickness of the muscular layer of these segments (Yasar and Forbes, 1999). In current study, we also found MIX group showed lower viscosity of jejunum. Thus, reduced muscular layer thickness in MIX group may due to the lower viscosity of gut contents.

*P. acidilactici* BCC-1 belongs to genus *Pediococcus*, but our microbiota data haven’t found *Pediococcus* in our BBC or MIX group. The previous study showed that Pediococcus only be detectable in chicks (3–4 days old) through 16S sequencing (Ocejo et al., 2019). Thus, the abundance of *Pediococcus* in our 21-day old chicken may lower than detection threshold. But the PCA analysis indicates XOS or BBC treatment dramatically altered gut microbiome. Compared with CT group, all 3 treatment groups (XOS, BBC and MIX) showed lower *Proteobacteria* and MIX also had lower *Campylobacter*. The phylum *Proteobacteria* contains many pathogens, including *Escherichia*, *Salmonella* and *Helicobacter* (Wu et al., 2020). *Campylobacter* is the leading causative agent of human bacterial enteritis and over 50% of poultry meat worldwide is contaminated with *Campylobacter* (Suzuki and Yamamoto, 2009). Thus, BBC and XOS treatment could improve poultry health and food safety by reducing the abundance of intestinal pathogenic bacteria.

Compared with the BBC group, the XOS treatment enriched *Barnesiella* and *Clostridium* XIVa. *Clostridium* XIVa contains a gene encoding butyryl CoA-acetyl CoA transferase and butyryl CoA-lactate transferase that can convert acetic acid and lactic acid to butyric acid (Van den Abbeele et al., 2013). In a mice study, supplementation of prebiotic milk oligosaccharides increased the abundance of *Barnesiella*, which could render the intestinal milieu less prone to inflammation (Weiss et al., 2014). Also, *Barnesiella* bacteria can eliminate and protect against intestinal antibiotic-resistant pathogenic bacteria in hospitalized patients (Ubeda et al., 2013). Therefore, increased abundance of *Clostridium* XIVa and *Barnesiella* may favor the SCFA production and enhance the anti-inflammatory capability of broilers. In microbial functional analysis, genes involved in transporters, ABC transporters were enriched in the XOS group. ABC transporters play crucial roles in the uptake of XOS (Ejby et al., 2013). Therefore, the increased genes related to ABC transporters may favor bacteria to utilize prebiotic XOS.

In contrast, *Parabacteroides*, *Anaerotruncus* and *Faecalibacterium* were enriched in the BBC group. *Parabacteroides* supplementation enhanced intestinal barrier integrity of mice (Koh et al., 2019) and attenuated experimental murine colitis through modulation of immunity (Kverka et al., 2011). Both *Anaerotruncus* and *Faecalibacterium* are butyrate-producing bacteria (Wang et al., 2018). Besides, *Faecalibacterium* is a critical player in the maintenance of intestinal and systemic host health (Rios-Covian et al., 2015). Thus, BBC supplementation potentially enhanced the gut barrier function and anti-inflammatory capability of broilers. Moreover, microbial genes in BBC group were mapped onto glycine, serine and threonine metabolism, vitamin B_6_ metabolism, cysteine and methionine metabolism, arginine and proline metabolism and fructose and mannose, which may favor the nutrients utilization of broilers.

The MIX group enriched *Lachnospiraceae* and *Clostridiaceae* XIVa when compared with BBC group. Compared with the XOS treated group, the MIX group enriched *Anaerotruncus*, *Parabacteroides* and *Clostridium* XIVb. Members of the *Lachnospiraceae* are able to utilize lactate and acetate to produce butyrate via the butyryl-CoA or acetate CoA transferase pathways or the butyrate kinase pathway (Flint et al., 2015). In addition to producing butyrate, *Lachnospiraceae* also plays a role in the biosynthesis of vitamin B_12_ and may exert a protective role in suppressing *Clostridium difficile* colonization in the gastrointestinal tract (Reeves et al., 2012). The microbial functional genes in the MIX group were involved in carbohydrate metabolism which could improve gut health and increase the biosynthesis of nutrients.

Both the XOS and BBC groups showed higher O-acetylserine 1 and pyridoxine levels than the control group. Pyridoxine is the cofactor cysteine metabolism and treatment with pyridoxine combined with folic acid normalized the serum homocysteine levels of hyperhomocysteinaemia patients (Buchel et al., 2005). Pyridoxine can also protect against cell injuries through its direct antioxidant activity (Roh et al., 2018). The gut microbiota is an essential source of B-vitamins and increase in pyridoxine in XOS and BBC groups may provide essential nutrient for the growth of broilers. Additionally, microbial functional genes showed both amino sugar and nucleotide sugar metabolism and galactose metabolism were enriched in the XOS and BBC groups. The amino sugar and nucleotide sugar metabolism product such as N-acetyl-D-glucosamine stabilizes the intestinal mucosal barrier by recovering intestinal epithelial cells and reducing gut permeability (Zhao et al., 2011). Both BBC and XOS group decreased concentrations of carbohydrate sorbitol. It indicates BBC and XOS may accelerate microbial fermentation. It has been reported that sorbitol supplementation results in selective enrichment of *Lactobacillus* and increased butyrate levels in rat intestine (Sarmiento-Rubiano et al., 2007). Therefore, supplementation with XOS could potentially improve amino acid metabolism and simultaneously curtail the growth of *Lactobacillus*.

The BBC group enriched 2-hydroxy-3-isopropylbutanedioic acid, (2*R*,3*S*)-2-hydroxy-3-isopropylbutanedioic acid and 3-(3-hydroxyphenyl) propionic acid and myo-inositol. Similarly, the MIX group enriched allo-inositol and 3-(3-hydroxyphenyl) propionic acid as well. Inositol is a growth factor for animals and microorganisms. It is able to form glucuronic acid via inositol oxidase, which is involved in the host sugar metabolism process and is further degraded to SCFA (Croze and Soulage, 2013). The 3-(3-hydroxyphenyl)-propionic acid can be produced by *Clostridium* bacteria (Rechner et al., 2004). Recently study found this microbial metabolite has a strong antioxidative capacity and can prevent Cd-induced biotoxicity (Cheng et al., 2021). The concentrations of 3-hydroxy-3-methylglutaric acid, succinic acid and multiple carbohydrates including sorbitol, xylose 1, D-arabitol and lyxose 1 in the BBC and MIX group were lower than CT group. The 3-hydroxy-3-methylglutaric acid promotes lipid and protein oxidative damage, reduces antioxidant defenses and impairs energy production in rats (Leipnitz et al., 2008; da Rosa et al., 2016). Gut microbiota-produced succinic acid has been shown to depress the proliferation rate of the epithelial cells in the colon, as well as reducing the crypt size (Inagaki et al., 2007). Succinate has been shown to accumulate in the large intestine of pigs with antibiotic-associated diarrhea (Tsukahara and Ushida, 2002). Therefore, decreased concentrations of 3-hydroxy-3-methylglutaric acid and succinate tend to prevent inflammation and maintain the gut health of broilers. Our previous study has been demonstrated *P. acidilactici* BCC-1 can effectively use xylose as an energy source (Lei et al., 2018) and other study found some *Pediococcus acidilactici* strains could utilize D-arabitol (Wang et al., 2019). Also, some *Pediococcus* species such as *Pediococcus parvulus* can effectively use sorbitol (Pérez-Ramos et al., 2017). Thus, BBC and MIX group may promote microbial carbohydrates fermentation. Compared with BBC group, MIX group has lower carbohydrates of fucose 1, sophorose 2 and isomaltose 2. It suggests combined supplementation of both XOS and BBC may have synergistic effect to further improve the microbial fermentation of carbohydrate.

The metabolite 4-hydroxyphenylpyruvate 1, a precursor of tyrosine (Klein et al., 2016) was enriched largely in the XOS and MIX group. Concomitantly, metabolome results showed both XOS and MIX group enriched in tyrosine metabolism pathway. The exact role of 4-hydroxyphenylpyruvate 1 is not well-known, but recently clinical study found baseline plasma levels 4-hydroxyphenylpyruvate were associated with a beneficial response on fecal microbiota transplantation (de Groot et al., 2020). The 4-hydroxyphenylpyruvate can be reduced to p-hydroxyphenyllactic acid by several beneficial bacteria such as *Lactobacillus* (González et al., 2017). In our study, the level of DL-p-hydroxyphenyllactic acid in MIX group was 7.4-fold higher than XOS group. It indicates BBC may have the ability to transform 4-hydroxyphenylpyruvate to hydroxyphenyllactic acid. The p-hydroxyphenyllactic acid has already been identified as an antioxidant compound by a radical-scavenging assay (Suzuki et al., 2013). Thus, compared with the sole addition of XOS, combined supplementation of both XOS and BBC may have synergistic effect to improve antioxidative ability of broilers.

## Conclusion

**FIGURE 5:**
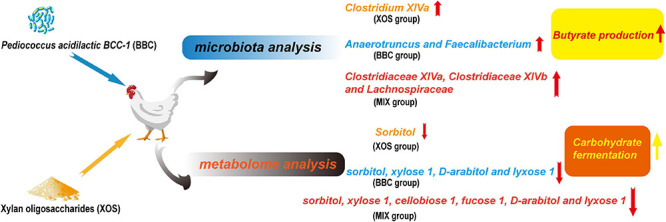
Graphic abstract of the influence of XOS and BBC treatment on cecal microbiota and metabolome of broilers.

In summary, both XOS and BBC can improve the feed efficiency of broilers. Microbiota and metabolome analysis showed combined supplementation of XOS and BBC decreased pathogenic bacteria, increased butyrate bacteria and promotes carbohydrate fermentation. Thus, XOS and BBC may have a synergistic role and combined supplementation of XOS and BBC may gain advantages of both XOS and BBC, which improve the performance of broilers by modulating gut microbiota and metabolome (Figure 5).

## Data Availability Statement

The datasets presented in this study can be found in online repositories. The names of the repository/repositories and accession number(s) can be found below: https://www.ncbi.nlm.nih.gov/, PRJNA592830.

## Ethics Statement

The animal study was reviewed and approved by the animal experiments involved in this study were approved by the China Agriculture University Animal Care and Use Committee in Beijing, China.

## Author Contributions

JY and ZL designed this research. YWu, ZL, YWa, and DY conducted the experiments. YWu, ZL, and YWa analyzed the data and wrote the draft of the manuscript. JY, SA, and YG checked and revised the manuscript. All authors contributed to the article and approved the submitted version.

## Conflict of Interest

The authors declare that the research was conducted in the absence of any commercial or financial relationships that could be construed as a potential conflict of interest.

## Footnotes

1 http://www.genome.jp/kegg/

2 http://www.nist.gov/index.html

3 http://www.metaboanalyst.ca

4 http://greengenes.lbl.gov

5 http://qiime.org

## Supplementary Material

Supplementary Figure 1 LEfSe (Linear discriminant analysis effect size) analysis of functional pathways of broiler cecum between XOS and control groups **(A)**; between BBC and control groups **(B)**; between MIX and control groups **(C)**.
