# Supplementary figures and images for: Metabolome and Microbiota Analysis Reveals the Conducive Effect of Pediococcus acidilactici BCC-1 and Xylan Oligosaccharides on Broiler Chickens
Source: Front Microbiol. 2021 May 28;12:683905. doi: 10.3389/fmicb.2021.683905 (PMC8192963; doi:10.3389/fmicb.2021.683905)

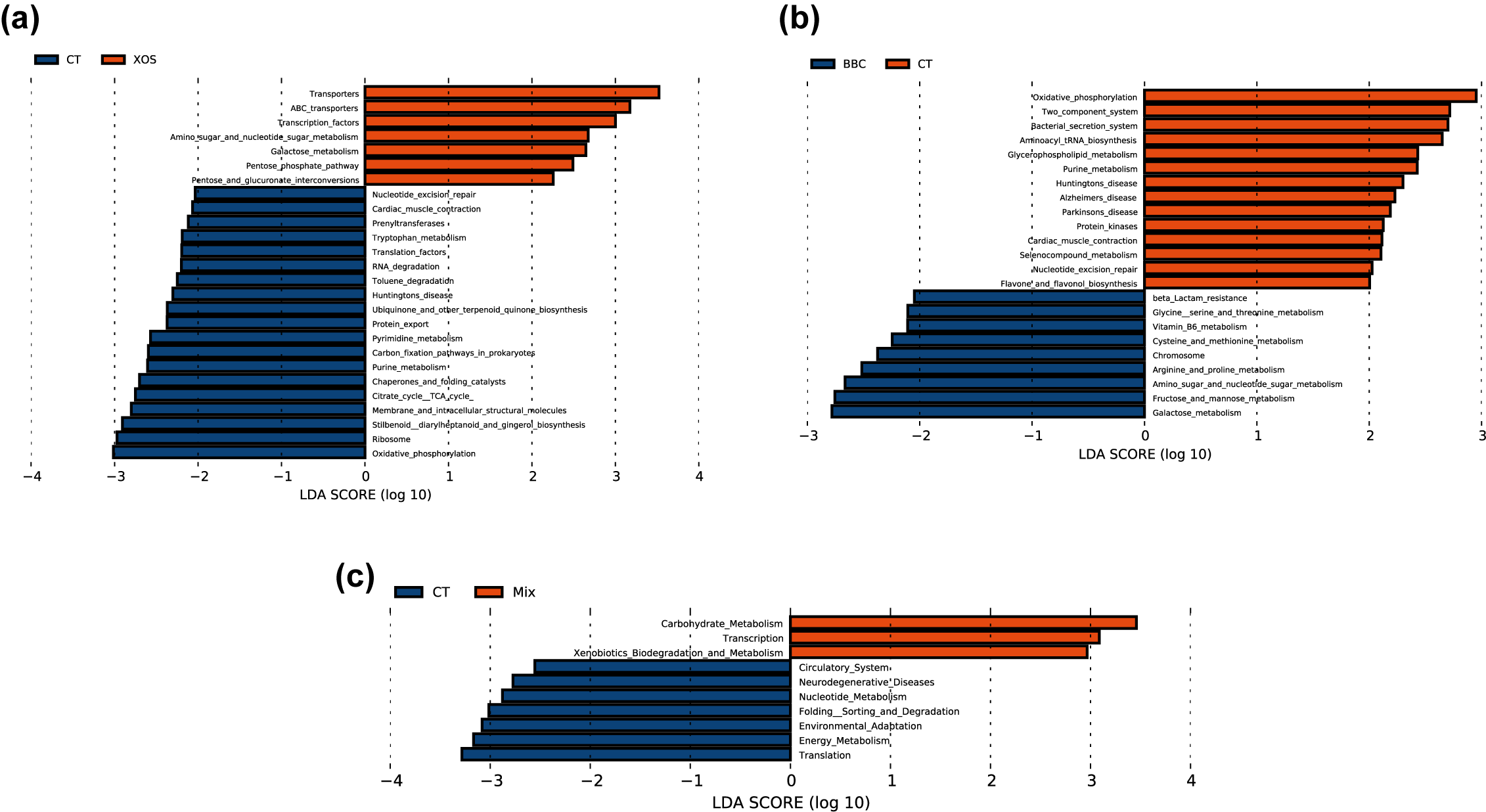

Supplement: Supplementary Figure 1 — LEfSe (Linear discriminant analysis effect size) analysis of functional pathways of broiler cecum between XOS and control groups (A); between BBC and control groups (B); between MIX and control groups (C). [file Image_1.TIF]
