# Supplementary material for: Metabolome and Microbiota Analysis Reveals the Conducive Effect of Pediococcus acidilactici BCC-1 and Xylan Oligosaccharides on Broiler Chickens
Source: Front Microbiol. 2021 May 28;12:683905. doi: 10.3389/fmicb.2021.683905 (PMC8192963; doi:10.3389/fmicb.2021.683905)
Supplement: Supplementary file 2 [file Data_Sheet_1.docx]

**Supporting information**

**Tables**

Table S1. Differential metabolites in XOS and CT group

| Metabolites | VIP | *P* value | Fold Change |
| --- | --- | --- | --- |
| 4-hydroxyphenylpyruvate 1 | 1.63 | 0.035 | 26.02 |
| O-acetylserine 1 | 2.42 | 0.019 | 4.05 |
| DL-dihydrosphingosine | 1.52 | 0.012 | 4.01 |
| Glycine-proline | 1.72 | 0.014 | 3.18 |
| Cis-gondoic acid | 1.33 | 0.038 | 2.93 |
| Pyridoxine | 1.54 | 0.003 | 2.64 |
| DL-p-Hydroxyphenyllactic acid | 1.44 | 0.034 | 0.28 |
| Creatine | 1.61 | 0.028 | 0.19 |
| Palmitoleic acid | 2.26 | 0.013 | 0.14 |
| Gluconic acid 1 | 1.90 | 0.023 | 0.14 |
| Sorbitol | 2.02 | 0.017 | 2.65E-07 |
| Salicylaldehyde | 2.02 | 0.030 | 2.37E-07 |

VIP = Variable Importance in Projection; *P* value calculated by Student's t test; Fold Change≥2 or ≤0.3.

If Fold change > 1, it means that this metabolite is higher in the XOS group than that in the CT group; Vice versa.

Table S2. Differential metabolites in BBC and CT group

| Metabolites | VIP^b^ | *P* value | Fold Change |
| --- | --- | --- | --- |
| 2-hydroxy-3-isopropylbutanedioic acid | 1.36 | 0.022 | 12.32 |
| Myo-inositol | 1.21 | 0.017 | 4.32 |
| Purine riboside | 1.07 | 0.025 | 4.06 |
| Elaidic acid | 1.25 | 0.012 | 3.99 |
| (2R,3S)-2-hydroxy-3-isopropylbutanedioic acid | 1.13 | 0.004 | 3.64 |
| 3-(3-hydroxyphenyl) propionic acid | 1.64 | 0.004 | 2.72 |
| Pyridoxine | 1.54 | 0.003 | 2.64 |
| O-acetylserine 1 | 1.47 | 0.009 | 1.99 |
| 4-Methyl-5-thiazolethanol | 1.39 | 0.018 | 0.30 |
| Thymidine 1 | 1.15 | 0.022 | 0.18 |
| N-Acetyl-beta-D-mannosamine 3 | 1.36 | 0.016 | 0.18 |
| Xanthine | 1.53 | 0.016 | 0.18 |
| P-cresol | 1.31 | 0.017 | 0.13 |
| 2-deoxy-D-glucose 2 | 1.27 | 0.006 | 0.12 |
| Oleic acid | 1.95 | 0.040 | 0.11 |
| Cis-Phytol | 2.48 | 0.0002 | 0.10 |
| Xylose 1 | 2.21 | 0.029 | 4.61E-07 |
| Sorbitol | 1.61 | 0.017 | 3.13E-07 |
| Salicylaldehyde | 1.61 | 0.030 | 2.80E-07 |
| Palmitoleic acid | 2.44 | 0.005 | 2.48E-07 |
| 2-Deoxyerythritol | 2.01 | 0.003 | 2.08E-07 |
| Gluconic acid 1 | 1.78 | 0.009 | 1.84E-07 |
| D-Arabitol | 2.45 | 0.012 | 1.23E-07 |
| Lyxose 1 | 1.74 | 0.028 | 1.04E-07 |
| 3-hydroxy-3-methylglutaric acid | 2.20 | 0.035 | 7.10E-08 |
| 3,4-dihydroxybenzoic acid | 2.51 | 5.32E-05 | 6.99E-08 |
| Citramalic acid | 1.99 | 0.009 | 4.82E-08 |
| Succinic acid | 1.88 | 0.030 | 2.20E-08 |

VIP = Variable Importance in Projection; *P* value calculated by Student's t test; Fold Change≥2 or ≤0.3

If Fold change > 1, it means that this metabolite is higher in the BBC group than that in the CT group; Vice versa.

Table S3. Differential metabolites in MIX and XOS group

| Metabolites | VIP | *P* value | Fold Change |
| --- | --- | --- | --- |
| 5-Hydroxyindole-3-acetic acid | 3.05 | 0.0001 | 3186861.16 |
| 2-Butyne-1,4-diol | 1.82 | 0.023 | 381105.22 |
| DL-p-Hydroxyphenyllactic acid | 2.31 | 0.009 | 7.41 |
| N-Ethylglycine 2 | 1.79 | 0.003 | 2.52 |
| 3-(4-hydroxyphenyl) propionic acid | 1.82 | 0.003 | 2.42 |
| Inosine | 1.69 | 0.023 | 2.34 |
| 2-ketoadipate 3 | 1.13 | 0.002 | 2.02 |
| 3-(3-hydroxyphenyl) propionic acid | 1.75 | 0.010 | 1.98 |
| Fucose 1 | 2.32 | 0.004 | 0.27 |
| DL-dihydrosphingosine 1 | 1.61 | 0.003 | 0.10 |
| D-erythro-sphingosine 2 | 1.65 | 0.033 | 0.08 |
| Stearic acid | 2.42 | 0.007 | 0.06 |
| 4-hydroxyphenylacetic acid | 2.19 | 0.015 | 0.03 |
| Oleic acid | 2.49 | 0.005 | 2.85E-07 |
| 2-Deoxyerythritol | 1.71 | 0.043 | 2.50E-07 |
| Cis-Phytol | 2.14 | 0.004 | 1.69E-07 |
| Acetol 5 | 3.03 | 0.021 | 1.43E-07 |
| 3,4-dihydroxybenzoic acid | 2.11 | 0.007 | 4.72E-08 |
| Pentadecanoic acid | 2.01 | 0.007 | 8.86E-09 |

VIP = Variable Importance in Projection; *P* value calculated by Student's t test; Fold Change>2

If Fold change > 1, it means that this metabolite is higher in the MIX group than that in the XOS group; Vice versa.

Table S4. Differential metabolites in MIX and BBC group

| Metabolites | VIP | *P* value | Fold Change |
| --- | --- | --- | --- |
| Gluconic acid 1 | 1.82 | 0.014 | 2,864,826 |
| Xanthine | 1.20 | 0.006 | 4.17 |
| Pantothenic acid | 1.32 | 0.018 | 3.87 |
| Urea | 1.58 | 0.003 | 3.27 |
| Palatinitol 2 | 1.64 | 0.027 | 3.23 |
| 3-Aminoisobutyric acid | 2.01 | 0.011 | 3.20 |
| Lactamide | 1.34 | 0.001 | 2.80 |
| 2-hydroxyvaleric acid | 1.46 | 0.001 | 2.57 |
| Proline | 1.91 | 0.015 | 2.35 |
| L-malic acid | 1.22 | 0.035 | 2.30 |
| Methionine 1 | 1.49 | 0.024 | 2.27 |
| N-Ethylglycine 2 | 1.10 | 7.19E-05 | 2.25 |
| 3-(3-hydroxyphenyl) propionic acid | 1.52 | 0.005 | 2.11 |
| Isoleucine | 1.76 | 0.007 | 2.02 |
| Glycolic acid | 2.19 | 0.0003 | 1.98 |
| Trans-4-hydroxy-L-proline 2 | 1.35 | 0.004 | 0.26 |
| Tagatose 1 | 1.87 | 0.006 | 0.24 |
| Threitol | 1.49 | 0.006 | 0.24 |
| Myo-inositol | 1.40 | 0.014 | 0.20 |
| Conduritol b epoxide 2 | 1.08 | 0.007 | 0.19 |
| Fucose 1 | 1.63 | 0.010 | 0.18 |
| Stearic acid | 2.52 | 4.39E-05 | 0.06 |
| Isomaltose 2 | 1.61 | 0.044 | 0.04 |
| Sophorose 2 | 1.30 | 0.036 | 0.04 |
| Pentadecanoic acid | 1.44 | 0.038 | 2.26E-08 |

VIP = Variable Importance in Projection; *P* value calculated by Student's t test; Fold Change>2

If Fold change > 1, it means that this metabolite is higher in the MIX group than that in the BBC group; Vice versa.
